# Clinical Characteristics and Prognostic Factors of Patients With Stenotrophomonas maltophilia Pneumonia: 10-Year Experience From a Single Center

**DOI:** 10.7759/cureus.47187

**Published:** 2023-10-17

**Authors:** Deniz Kızılırmak, Yavuz Havlucu

**Affiliations:** 1 Department of Chest Diseases, Manisa Celal Bayar University, Manisa, TUR

**Keywords:** mortality, clinical characteristics, pneumonia, infection, stenotrophomonas maltophilia

## Abstract

Introduction: Stenotrophomonas maltophilia infection is gaining importance as an important cause of nosocomial pneumonia. S. maltophilia infection occurs especially in patients with a history of immunosuppression, comorbidity, or multiple antibiotherapy uses. A retrospective 10-year study was carried out to determine the clinical characteristics of all patients with S. maltophilia pneumonia, antibiotic resistance pattern, and risk factors associated with hospital mortality.

Materials and methods: Hospitalized pneumonia patients with S. maltophilia culture positivity were identified, and their medical records were reviewed. Risk factors associated with hospital mortality were analyzed. Any variable with a significant association with mortality in the univariate analysis was entered in a multivariate forward stepwise logistic regression model to identify independent risk factors for death.

Results: Seventy-two patients (mean age: 67.3 years, 65.2% males) with S. maltophilia pneumonia were included in the study. All patients had at least one comorbidity. The most common comorbidities were chronic obstructive pulmonary disease, diabetes mellitus, chronic renal failure, malignancy, and cardiac diseases. Percentage resistance to trimethoprim-sulfamethoxazole (5.5%) was lower than that for fluoroquinolones (12.5%). By using multivariate analysis, respiratory insufficiency needed mechanical ventilation, low hemoglobin level, age>65 years, previous antibiotic usage, and hypotension were the independent prognostic factors for mortality.

Conclusion: S. maltophilia is emerging as an important pathogen with an increased risk of mortality in patients with respiratory insufficiency who need mechanical ventilation, a low hemoglobin level, >65 years of age, previous antibiotic usage, and hypotension. Empiric therapy should include agents active against S. maltophilia, such as newer fluoroquinolones and trimethoprim-sulfamethoxazole.

## Introduction

Stenotrophomonas maltophilia (S. maltophilia) is a gram (-), non-fermentative bacillus that is frequently encountered as the causative agent of hospital-acquired infections [1]. It can cause infections such as pneumonia, skin and soft tissue infections, urinary tract infections, otitis, conjunctivitis, meningitis, endocarditis, and bacteremia [2]. S. maltophilia infection, which poses a risk especially for patients on immunosuppression and ventilator support, is an important cause of nosocomial pneumonia due to its characteristic natural resistance to many broad-spectrum antibiotics, such as broad-spectrum penicillins and aminoglycosides. Trimethoprim-sulfomethoxazole and fluoroquinolones are frequently preferred in treatment [3]. In S. maltophilia infections, in addition to natural resistance, there is acquired resistance obtained through plasmids, transposons, and integrons [4,5].

S. maltophilia is among the 10 most common hospital-associated pneumonia agents in Europe and the United States [6]. In clinical studies, mortality rates associated with S. maltophilia infections range between 21% and 69% [2]. Reasons such as prolonged use of broad-spectrum antibiotics, immunosuppression, prolonged ventilator support, severe underlying disease, chronic lung disease, and tracheostomy are risk factors for S. maltophilia-associated pneumonia [7]. In some cases, a second agent other than S. maltophilia can be isolated, and it may become difficult to control the infection. For these reasons, S. maltophilia pneumonia is a difficult-to-treat disease that significantly affects mortality, especially in patients with a severe course, and can be confused with other diagnoses [8].

The clinical features, resistance pattern, and mortality-related risk factors of S. maltophilia pneumonia are very important in terms of starting the right treatment selection and reducing mortality in the early period. In this study, which retrospectively examined 10-year data from a single center, the clinical characteristics, antibiotic resistance patterns, and risk factors associated with hospital mortality in hospitalized patients with S. maltophilia pneumonia were investigated.

## Materials and methods

This study, which included data from a single center, was designed as a retrospective analysis. The medical records of patients diagnosed with S. maltophilia pneumonia at a university hospital between July 2010 and June 2020 were reviewed, and risk factors associated with hospital mortality were analyzed. Patients who were hospitalized with a diagnosis of pneumonia and had S. maltophilia growth in the microbiological examination of respiratory material were included in the study. The inclusion criteria for the study were being over the age of 18, being hospitalized with a diagnosis of pneumonia, and having S. maltophilia growth in the microbiological examination of the respiratory material sample taken during this period. Exclusion criteria were considered to be being under 18 years of age, having a diagnosis of cystic fibrosis, having incomplete hospital records, and missing data required for the statistical evaluation of the study. Ethics committee approval was received from the Health Sciences Ethics Committee to conduct the study (decision date: July 22, 2020, decision number: 20.478.486/455).

The gender of the patients, their age at the time they were followed up with S. maltophilia pneumonia, average hospitalization days, vital signs, laboratory parameters, comorbidities, and mortality-related conditions were recorded. In addition, the day of hospitalization before S. maltophilia infection, antibiotic use in the last 30 days, surgical procedure, steroid or immunosuppressive agent use, and other isolated microorganisms were recorded. Clinical and laboratory risk factors for hospital mortality due to S. maltophilia pneumonia were analyzed.

The data obtained in the study were evaluated statistically with IBM SPSS Statistics for Windows, Version 21 (Released 2012; IBM Corp., Armonk, New York, United States). As descriptive statistics, frequency, percentage values, median (interquartile range), mean, and standard deviation values were determined. The analyzed variables followed a normal distribution. Therefore, Student's t-test and one-way analysis of variance (ANOVA) were used for numerical variables. Comparisons between categorical variables were made with the chi-square test. Comparative correlation analyses (Pearson) were performed to determine the variables affecting mortality. Any variable with a significant association with mortality on univariate analysis was analyzed according to a multivariable forward stepwise logistic regression model to be identified as an independent risk factor for mortality. In statistical evaluations, p<0.05 was considered statistically significant.

## Results

Seventy-two patients with S. maltophilia pneumonia, not related to cystic fibrosis, were included in the study. The mean age of the patients was 65.3±12.9 years, and 47 (65.2%) were male. The average hospital stay of the patients was 23.7±17.4 days. A total of 76 samples obtained from 72 patients were culture-positive. The 72 patients were hospitalized in medical (61.1%), surgical (5.5%), and hematology/oncology departments (8.3%) or intensive care units (25.1%). All patients had at least one comorbidity. The most common comorbidities were cardiac diseases, chronic obstructive pulmonary disease, diabetes mellitus, malignancies, chronic renal failure, and rheumatological diseases (Table 1). Three of the malignant patients had hematologic malignancies, and the others had solid organ malignancies. There was no lung involvement in any of the patients with rheumatological diseases.

**Table 1:** Demographics and basic characteristics of the patients SD: Standard deviation, n: number, COPD: chronic obstructive pulmonary disease.

Age, years (mean±SD)	67.3±12.9
Gender (n, %)	
Female	25 (34.8)
Male	47 (65.2)
Average hospitalization days (mean±SD)	23.7±17.4
Comorbidities (n, %)	
Any cardiac disease	59 (81.9)
COPD	48 (66.7)
Diabetes mellitus	37 (51.3)
Malignancy	19 (26.4)
Chronic renal failure	15 (20.8)
Rheumatological disease	12 (16.7)

The patients had an average hospital stay of 10.9±8.5 days before S. maltophilia pneumonia. Forty-one (56.9%) of the patients had received antibiotic treatment, and 19 (26.4%) had received systemic steroid or immunosuppressive treatment in the last 30 days. Six patients (8.3%) had a history of surgery 30 days before the culture positivity. In 67 (93.1%) of the patients with S. maltophilia pneumonia, a pathogen other than S. maltophilia was grown in culture. Acinetobacter baumannii was the most frequently isolated pathogen in the respiratory samples of 35 patients (48.6%), followed by S. maltophilia pneumonia. Other frequently occurring pathogens were Pseudomonas aeruginosa in 29 (40.3%) patients and Klebsiella pneumoniae in 21 (29.2%) patients in the respiratory samples of patients with S. maltophilia pneumonia. Characteristic features at the beginning of the S. maltophilia infection are presented in Table 2.

**Table 2:** Characteristics of patients at the onset of S. maltophilia infection SD: Standard deviation, n: number.

Hospitalization days prior to S. maltophilia infection, (mean±SD)	10.9±8.5
Antibiotic treatment in past 30 days, (n, %)	41 (56.9)
Surgical procedure in past 30 days, (n, %)	6 (8.3)
Steroids or immunosuppressors in past 30 days, (n, %)	19 (26.4)
Other microorganisms isolated from patients (n, %)	67 (93.1)
Acinetobacter baumannii	35 (48.6)
Pseudomonas aeruginosa	29 (40.3)
Klebsiella pneumoniae	21 (29.2)
Escherichia coli	14 (19.4)
Staphylococcus aureus	13 (18.1)
Any fungal organisms	25 (34.7)
Others	19 (26.4)

Trimethoprim-sulfamethoxazole resistance was found in four patients (5.5%), whereas fluoroquinolone resistance was found in 9 patients (12.5%). During their hospital stay, 17 patients (23.6 percent) died. By using multivariate analysis, the independent prognostic factors for mortality were respiratory insufficiency requiring mechanical ventilation, low hemoglobin level (10 g/dL), age over 65 years, previous antibiotic use, and systolic peripheral arterial pressure less than 100 mmHg (Table 3).

**Table 3:** Risk of death from S. maltophilia pneumonia OR: Odds ratio, CI: confidence interval, mmHg: millimeters of mercury, dL: deciliters.

Risk factors	OR	95% CI	p
Respiratory insufficiency requiring mechanical ventilation	5.79	2.47-11.35	<0.05
Age>65 years	3.49	1.93-10.72	<0.05
Presence of malignancy	2.37	0.91-4.63	>0.05
Antibiotic treatment in past 30 days	6.81	3.59-15.04	<0.05
Surgical procedure in past 30 days	1.94	0.57-2.41	>0.05
Steroids or immunosuppressors in past 30 days	1.47	0.73-2.05	>0.05
Hospitalization days prior to S. maltophilia infection	1.52	1.20-1.97	<0.05
Systolic blood pressure<100mmHg	2.05	1.64-4.27	<0.05
Hemoglobin level<10 g/dL	2.41	1.52-4.83	<0.05

## Discussion

In our study, S. maltophilia pneumonia was mostly diagnosed in hospitalized patients, the mean age of the patients was high, and all patients had comorbid diseases. Additionally, the patients had an average of 10 days of hospitalization history before S. maltophilia pneumonia. S. maltophilia is an important cause of infection and mortality in immunosuppressed patients, intensive antibiotic use, mechanical ventilation, and hospitalized patients, and determining risk factors is important for the management of the infection. These results obtained from our study show that caution should be taken in terms of S. maltophilia pneumonia in people of advanced age and with comorbid diseases who are hospitalized and treated, and prolonged hospitalization should be avoided as much as possible in these patients.

In previous studies, underlying malignancy, the presence of a permanent device such as a catheter, chronic respiratory disease, immunosuppression, previous antibiotic use, and long-term hospital or intensive care stay have been shown to be among the risk factors for S. maltophilia infection [9,10]. In our study, the long hospital stays in patients with S. maltophilia growth in microbiological culture, the high rate of antibiotic use in the last 30 days, and the history of immunosuppressive agent use in approximately one-quarter of the patients are compatible with the presence of previously identified risk factors.

In a study evaluating the characteristics of S. maltophilia bacteremia in 48 patients in Taiwan, intensive care unit stays, mechanical ventilator use, and central venous catheter use were found to be risk factors associated with mortality in patients with S. maltophilia bacteremia [11]. Similar to this study, our study included respiratory failure requiring mechanical ventilator support among the risk factors associated with mortality.

Publications about S. maltophilia pneumonia are often in the form of case series. There are not many studies with large series. In the study of Gozel et al., pneumonia was detected in 50.7% of 71 cases followed in the intensive care unit with S. maltophilia growth, and in multivariate analysis, inappropriate antibiotic use and the presence of multiple organ dysfunction syndrome were found to be independent risk factors for 14-day mortality [12]. According to the results of our study, using antibiotics in the last thirty days is among the risk factors associated with mortality. In our study, unlike previous studies, low hemoglobin levels (10 g/dL), being over 65 years of age, and systolic peripheral arterial pressure below 100 mmHg were also shown to be among the risk factors associated with mortality.

In the study conducted by Batra et al., the most frequently isolated pathogens in the respiratory samples of patients, followed by S. maltophilia pneumonia were Acinetobacter baumannii at a rate of 52% and Pseudomonas aeruginosa at a rate of 30% [13]. Similarly, in our study, the most frequently isolated pathogen was Acinetobacter baumannii with a rate of 48.6%, and the second most frequently isolated pathogen was Pseudomonas aeruginosa with a rate of 40.3%.

S. maltophilia is naturally resistant to most broad-spectrum antibiotics, including broad-spectrum beta-lactams, aminoglycosides, and carbapenems. S. maltophilia infections are difficult to treat due to their intense resistance to many antibiotics. Especially with the detection of S. maltophilia sensitivity and antibiotic resistance status in our region, early treatment can be arranged for patients at risk. In the study conducted by Hazırolan et al., covering an 8-year period and evaluating 195 S. maltophilia isolates, trimethoprim-sulfamethoxazole resistance was determined to be 4.08% on average, and levofloxacin resistance was determined to be 11.71% [14]. In our study, trimethoprim-sulfamethoxazole resistance was found to be 5.5% and fluoroquinolone resistance was found to be 12.5%, similar to the literature.

The main limitations of our study are that it is a retrospective study and cannot be generalized throughout the country due to its single-center nature. One of the limitations of the study is that the direct effects of S. maltophilia pneumonia could not be examined. This is because S. maltophilia infections are frequently accompanied by another pathogenic microorganism. More comprehensive studies investigating isolated S. maltophilia infections would be beneficial. The salient aspects of our study are that it covers a 10-year-long experience and evaluates patients from all in-hospital units, including surgery, hematology/oncology, and intensive care units.

## Conclusions

The results obtained from our study show that caution should be exercised in terms of S. maltophilia pneumonia in people who are hospitalized and elderly and have comorbid diseases. Because most patients have a co-infection with another pathogen, there is not enough information about isolated S. maltophilia pneumonia. The risk of mortality is high in patients with respiratory failure requiring mechanical ventilation, a low hemoglobin level, an age over 65 years, previous antibiotic use, and hypotension. Empiric treatment should include agents such as new types of fluoroquinolones and trimethoprim-sulfamethoxazole.
